# Impact of ozonated water disinfestation on soil fungal community composition in continuous ginger field

**DOI:** 10.1371/journal.pone.0266619

**Published:** 2022-04-07

**Authors:** Bo Zhang, Liguo Ma, Yueli Zhang, Kai Qi, Changsong Li, Junshan Qi

**Affiliations:** Institute of Plant Protection, Shandong Academy of Agricultural Sciences/Shandong Province Key Laboratory of Plant Virology, Jinan, Shandong, P. R. China; Universita degli Studi di Pisa, ITALY

## Abstract

This study aimed to explore the impact of ozonated water (OW) disinfestation on soil fungal community composition in continuous ginger field. All soil samples were collected in continuous ginger field. There were two groups and 5 time points (0, 1, 3, 5, 9 day) in our study, including OW disinfestation treatment group (O_3_ group) and control group (CK group). Via internal transcribed spacer (ITS) sequencing and further analysis, the changes of fungal community composition were determined. As a result, at 0 and 9 days after aeration, the operational taxonomic units (OTUs) in O_3_ group were significantly higher than that in CK group. Compared with the CK group, in O_3_ group: the ACE and Chao1 index significantly increased on day 1, and the Shannon index significantly decreased while Simpson index significantly increased on day 0 after aeration. In O_3_ group, there were dynamic changes of top 10 abundance fungi from the genus-level and the growth of *Trichoderma* and *Rhodotorula* had been promoted while *Hannaella* was inhibited. In conclusion, OW disinfestation had complicated impacts on fungal communities in continuous ginger fields. The growth of *Trichoderma* and *Rhodotorula* has been promoted during disinfestation, which provided more reference information for soil OW disinfestation research.

## 1 Introduction

In recent decades, many countries are facing the problem of limited arable land all over the world [1]. Subsequently, intensive cultivation like continuous monocropping and overfertilization has been widely used in order to meet the increasing demands [1,2]. Long-term continuous cropping often results in plant pathogen enrichment and imbalanced microbial community in soil [3,4], which is one of the main reasons for the serious yield decline [2]. Numerous studies have demonstrated that microorganisms could play an indispensable role not only in soil healthy [2] but also in plant health and growth [1]. In addition, fungus is one of the most abundant microorganisms in soil, which plays an important part in nutrient recycling in terrestrial ecosystems [5,6]. It has been widely recognized that soil fungi can impact various soil biogchemical processes [7]. Some microorganisms could improve the quality and crop yield of the soil through organic matter degradation and transformation [8,9]. Therefore, it is quite important for planting industry to protect the balanced soil microbial community and soil ecosystem.

In order to control soil-borne diseases and support high yields, preplant soil disinfestation is usually used in most intensive cropping systems [1]. Ozone (O_3_) is a recognized broad-spectrum and environmentally friendly insecticide [10,11], which has been applied to several fields like plant diseases control in facility agriculture [12]. Ozone is a powerful oxidant and ozonated water (OW) usually has stronger oxidation. OW is unstable, which decomposes rapidly into oxygen and would not leave toxic residues [13]. Thus, it is an environment-friendly way leading to less influence on soil microbial community. At present, some studies have focused on the effects of OW disinfestation on crops [12,13], but few studies paid attention to it’s influence on soil microbial community as far as we know. However, the negative impact of soil disinfestation on soil microbial community is a controversial problem.

Accordingly, in this study, the continuous ginger fields treated with OW were compared with untreated ginger fields. The changes of fungal community abundance, diversity and structure were then analyzed using internal transcribed spacer (ITS) sequencing in order to explore the impact of OW disinfestation on soil fungal community composition.

## 2 Materials and methods

### 2.1 Soil samples collection

Soil was collected from farms in Shandong, China. All soil samples were collected in continuous ginger field, which had been used for many years with no disinfestation, the ginger field had serious soil diseases. Soil was randomly sampled at five points on diagonal line and samples were collected from the top 25 cm of soil, which were packed in self-sealing bags and were labeled well for future usage. Samples were collected at 0, 1, 3, 5 and 9 days after the OW treated, respectively, including the control group (CK group) samples and the OW disinfestation treatment group (O_3_ group) samples.

Our sequence data has been uploaded to the National Center for Biotechnology Information (NCBI) (https://www.ncbi.nlm.nih.gov/) database, and the accession number is PRJNA746256.

### 2.2 DNA extraction

MoBio Powersoil^®^ DNA Isolation Kit (MoBio Laboratories, USA) was used to extract the total genomic DNA from each soil sample. The DNA quality was determined by gel electrophoresis (1% agarose). The DNA concentration was determined by NanoDrop 1000 UV spectrophotometer (Thermo Scientific, USA).

### 2.3 High-throughput sequencing and bioinformatics analysis

The universal primers [5′-CTTGGTCATTTAGAGGAAGTAA-3′] and [5′-GCTGCGTTCTTCATCGATG-3′] were used to amplify fungal genes in ITS1 region. MiSeq sequencing was conducted on an Illumina^®^ MiSeq sequencer (Illumina, USA) by Shanghai Tianhao Biotechnology Co., Ltd. (Shanghai, China) and paired-end sequencing (2 × 250 bp) was used. The sequencing data was quality-filtered and merged to obtain high-quality reads using TrimGalore software and FLASH software [14]. UPARSE software was used to perform operational taxonomic unit (OTU) clustering on nonrepeating sequences according to 97% similarity [15]. Moreover, the chimeras were removed during the clustering process to obtain the representative sequence of OTUs. The Mothur software was used to compare each ITS representative sequence with those deposited in the Ribosomal Database Project (RDP) ITS database (http://rdp.cme.msu.edu/) [16] to determine the classification, based on a confidence threshold of 60%. The α diversity was calculated using Mothur software, including the Coverage index, ACE index, Chao1 index, Shannon index and Simpson index. QIIME software [17] was used to calculate the unweighted UniFrac value and Principal Coordinate Analysis (PCoA) was used to visualize changes of soil microbial communities. A linear discriminant analysis effect size (LEfSe) [18] was performed to find differential biomarkers between two groups using relative abundance.

### 2.4 Statistical analysis

Wilcoxon symbolic rank sum test (R software v3.6.2) was used to compare α diversity differences (ACE index, Chao1 index, Shannon index and Simpson index) among different groups and P < 0.05 was used as significance threshold. Wilcoxon symbolic rank sum test was used to compare the differences of microbial communities with top 10 genus abundance in soil. LEfSe analysis basing on Kruskal-Wallis sum-rank test determined the changes of the genus abundance between the OW treatment group and the control group. |LDA score|>2 and P<0.05 was used as the significance threshold.

## 3 Results

### 3.1 Changes in microbial community OTUs

Based on the genetic sequencing results of the soil microbial communities, a total of 10,724,257 effective reads were obtained and the average length of effective reads was 232.59 bp. Compared with the CK group, the shared OTUs in O_3_ group at 1, 3, 5 and 9 days after aeration was all more than that of shared OTUs at 0 day (S1 Fig). Additionally, at 0 and 9 days after aeration, the number of unique OTUs in O_3_ group was significantly higher than that in CK group (P < 0.001), which indicated that the fungal communities in soil were significantly different after the OW treatment, compared with the CK group.

### 3.2 Changes of fungal abundance and diversity

**Fig 1:**
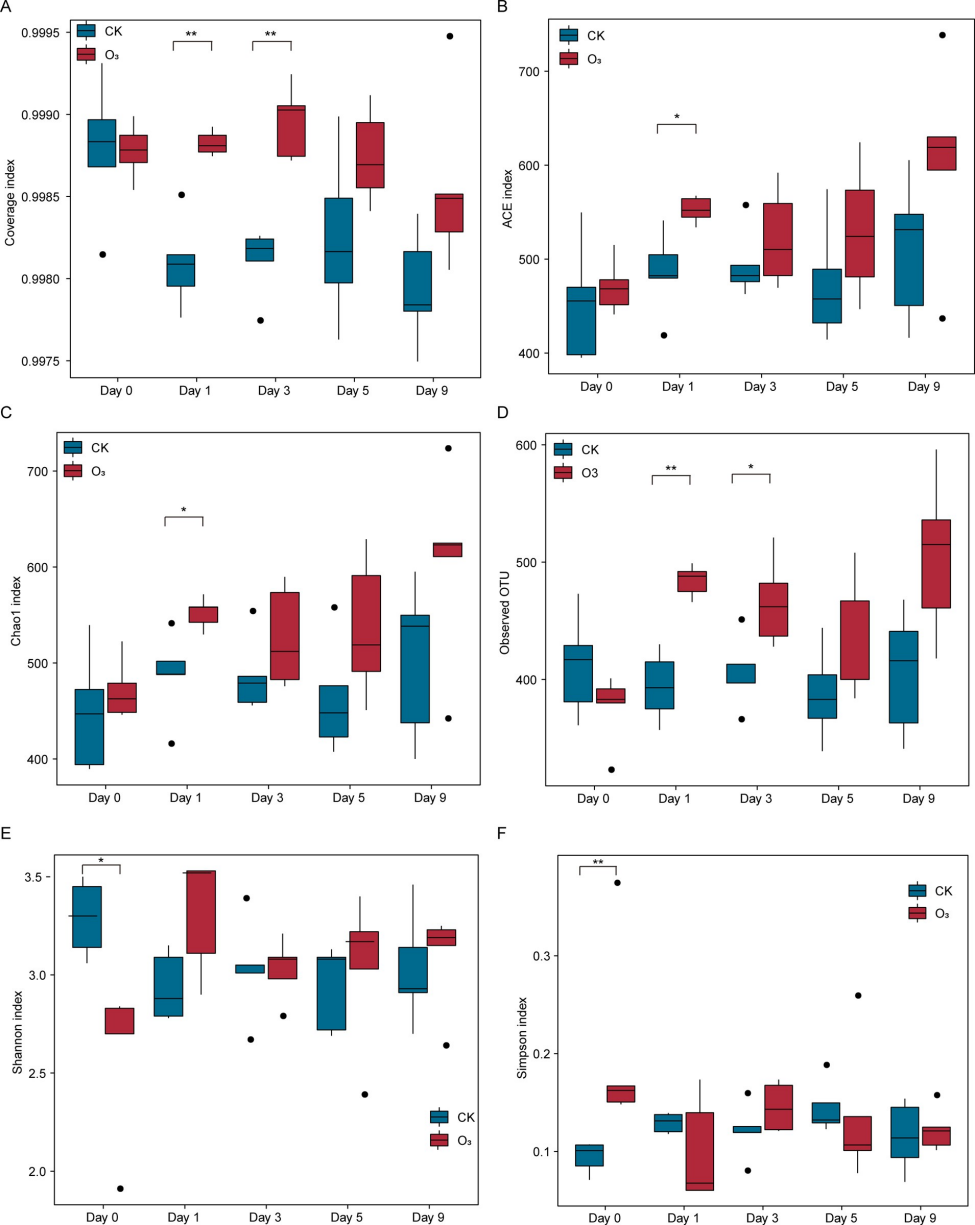
Microbial community diversity index of the CK group and O_3_ group, respectively. **(A)** Coverage index **(B)** ACE index **(C)** Chao1 index **(D)** Observed OTU **(E)** Shannon index **(F)** Simpson index. CK: Control group; O_3:_ The OW disinfestation treatment group. Statistical significance: * p < 0.05, ** p < 0.01, *** p < 0.001, **** p < 0.0001.

The dilution curve of samples (S2 Fig) suggested that the sampling and ITS genetic sequence data was sufficient to estimate the diversity of fungal communities. Coverage index showed that the sequencing coverage of samples was good (0.9975–0.9995) (Fig 1A). Based on 97% consistent sequence, 99% of the species presented in all samples. Compared with the CK group, the ACE and Chao1 index of the O_3_ group significantly increased (P < 0.05) at the first day after aeration (Fig 1B and 1C). During other time, there was no significant ACE and Chao1 index difference between the CK and O_3_ group. Compared with CK group, the OTU number in O_3_ group significantly increased at the first and third day, but there was no significant OTU number difference between the CK group and O_3_ group (Fig 1D). On day 0 in O_3_ group, the Shannon index significantly decreased while Simpson index increased significantly, but there was no significant difference between the CK group and O_3_ group in the following days (Fig 1E and 1F).

The results showed that the OW disinfestation transitorily stimulated the species abundance increasing and fungal diversity decreasing, however along with the extended time of aeration, species abundance and fungal diversity gradually returned to the untreated level.

### 3.3 Principal coordinate analysis (PCoA)

**Fig 2:**
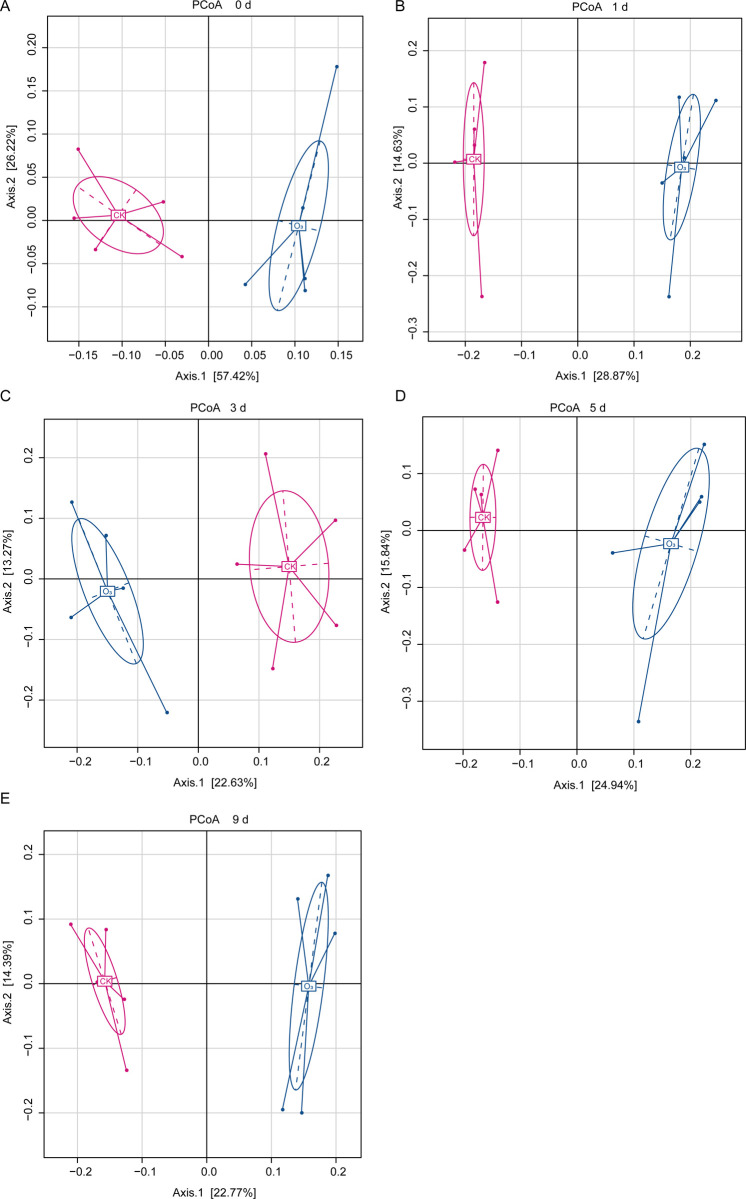
Microbial community principal coordinate analysis (PCoA) of the CK group and O_3_ group. (A-E) The PCoA results at 0, 1, 3, 5 and 9 days.

The PCoA results showed that the soil samples of two groups (CK and O_3_ group) were completely separated apart at 0, 1, 3, 5 and 9 days (Fig 2), which indicated that there was difference in soil samples between CK group and O_3_ group.

### 3.4 Changes of microbial communities with top 10 genus abundance in soil

**Fig 3:**
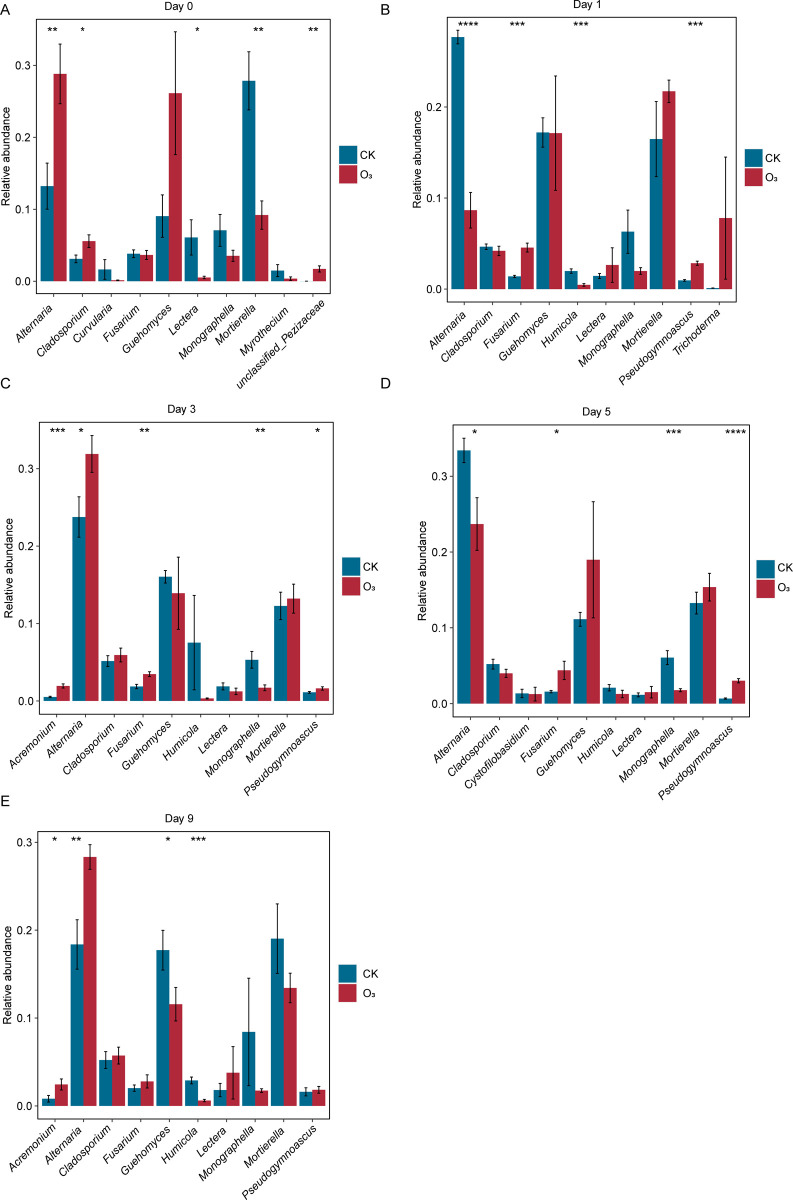
Changes of microbial communities with top 10 genus abundance in soil. (A-E) Changes of microbial communities at 0, 1, 3, 5 and 9 days. Statistical significance: * p < 0.05, ** p < 0.01, *** p < 0.001, **** p < 0.0001.

In all samples, *Alternaria*, *Mortierella*, *Guehomyces*, *Monographella* and *Cladosporium* were dominant fungi from the genus-level (S3 Fig). The fungal community changes of top 10 abundance from the genus-level between CK group and O_3_ group were further compared at various days. Compared with the CK group, *Alternaria* in O_3_ group significantly increased on the 0th, 3rd and 9th day and significantly decreased on the 1st and 5th day after aeration. *Cladosporium* significantly increased on day 0 and then returned to untreated level. *Lectera* and *Mortierella* significantly decreased on day 0 and then returned to untreated levels. *Fusarium* in O_3_ group was significantly higher than that in CK group during 1–5 days, but it decreased gradually along with time and returned to the untreated level on the 9th day. *Guehomyces* was relatively stable during the first 5 days and was significantly lower than that of the CK group on the 9th day. *Monographella* was significantly lower than that of the CK group during 3–5 days and returned to untreated levels on the 9th day. *Humicola*, *Pseudogymnoascus* replaced *Curvularia*, *Myrothecium* and *unclassified_Pezizaceae* and became the dominant fungal community (among top 10) after day 1. *Humicolain* in O_3_ group was significantly higher than that in CK group on day 1 and day 9. *Pseudogymnoascus* in O_3_ group was significantly higher than that in CK group during 1–5 days, but it returned to the untreated level on the 9th day (Fig 3).

### 3.5 Changes of biomarkers of soil fungal communities

**Fig 4:**
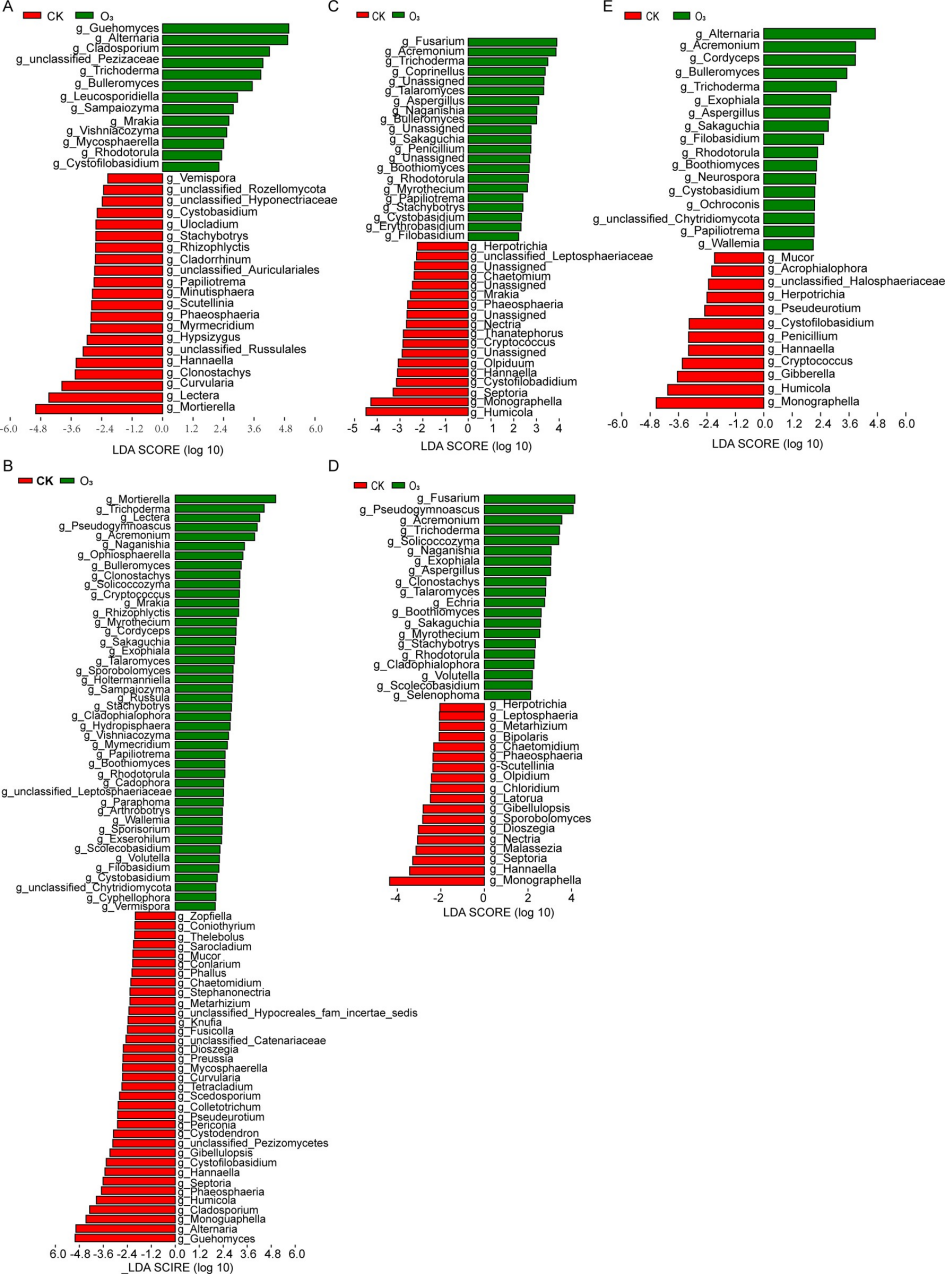
Significantly differential biomarkers between CK group and O_3_ group at various days were analyzed using LEFSe. (A-E) Differential biomarkers at 0, 1, 3, 5 and 9 days. Statistical significance: * p < 0.05, ** p < 0.01, *** p < 0.001, **** p < 0.0001.

Significantly different biomarkers between CK group and O_3_ group on various days were displayed using LEFSe analysis. The number of differential biomarkers between CK group and O_3_ group in the initial exposure stage (0–1 days) was gradually increasing and then decreased gradually during 1–9 days. Compared with the CK group, on day 0, *Guehomyces*, *Alternaria* and other 13 genera significantly increased in O_3_ group, and *Vermispora*, *Cystobasidium* and other 21 genera significantly decreased (Fig 4A). Compared with the CK group, on day 1, *Mortierella*, *Trichoderma* and other 44 genera significantly increased in O_3_ group, and *Coniothyrium*, *Knufia* and other 35 genera significantly decreased (Fig 4B). Compared with the CK group, on day 3, *Fusarium*, *Acremonium*, *Trichoderma* and other 21 genera significantly increased in O_3_ group, and *Herpotrichia*, *Cryptococcus* and other 18 genera significantly decreased (Fig 4C). Compared with the CK group, on day 5, *Fusarium*, *Pseudogymnoascus*, *Acremonium* and other 20 genera significantly increased in O_3_ group, and *Herpotrichia*, *Sporobolomyces* and other 17 genera significantly decreased (Fig 4D). Compared with the CK group, on day 9, *Alternaria*, *Acremonium*, *Cordyceps* and other 17 genera significantly increased in O_3_ group, and *Halosphaeriacea*, *Herpotrichia* and other 12 genera significantly decreased (Fig 4E).

Compared with the CK group, *Trichoderma* and *Rhodotorula* significantly increased in O_3_ group during 0–9 days, and *Boothiomyces*, *Sakaguchia*, *Acremonium* and *Aspergillus* also significantly increased during 3–9 days (S4A Fig). Compared with the CK group, *Hannaella* significantly decreased in O_3_ group during 0–9 days, and *Herpotrichia* and *Monographella* also significantly decreased during 3–9 days (S4B Fig). The results showed that OW disinfestation had promoted the growth of *Trichoderma* and *Rhodotorula*, but it had inhibited the growth of *Hannaella*.

## 4 Discussion

In this research, via the comparison of the continuous ginger fields treated with OW and untreated ginger fields, we explored the the influence of OW disinfestation on soil fungal community composition. The OW disinfestation transitorily stimulated the fungus abundance increasing and diversity decreasing and there were dynamic changes of top 10 abundance fungi from the genus-level. The OW disinfestation had promoted the growth of *Trichoderma* and *Rhodotorula*, but it had inhibited the growth of *Hannaella*.

Recently, ozone has been gradually recognized as a powerful and effective oxidant for water treatment [19]. Moreover, aqueous ozone treatments have been recently evidenced to reduce contaminant colony forming units (CFUs) [20], which makes OW disinfestation more meaningful. Thus, we expected to explore its potential influence on fungal community in field. A research in long-term continuous soybean field has reported that long-term continuous cropping led to the trends of fungal community development to antagonistic to plant health [21], whose similar situation supported our observation in continuous ginger field. Firstly, we have investigated the changes of fungal abundance and diversity in soil after treated with OW. The number of unique OTUs in O_3_ group (OW treated group) was significantly higher than that in CK group at 0 and 9 days aeration, which indicated that there were more fungi in soil in O_3_ group at 0 and 9 days after aeration. As for fungal diversity, compared with the CK group, the ACE and Chao1 index of the O_3_ group significantly increased at the first day after aeration. On day 0 in O_3_ group, the Shannon index significantly decreased while Simpson index increased significantly. Previous researches have evidenced that Simpson index, Shannon index, ACE index and Chao1 index were reliable indicators of fungal diversity [21]. Our findings suggested that the OW disinfestation transitorily stimulated the fungal abundance increasing and fungal diversity decreasing, however along with the extended time of aeration, fungal abundance and diversity gradually returned to the untreated level. Moreover, it has been demonstrated that soil fungal diversity was closely associated with the plant health and soil-borne diseases [22,23]. Collectively, the OW disinfestation would transitorily impact the fungal abundance and diversity, which might be benefit for fungal restoration after disinfestation.

Additionally, various fungal community compositions at genus level and their abundance were then analyzed. In all samples, *Alternaria*, *Mortierella*, *Guehomyces*, *Monographella* and *Cladosporium* were dominant fungi. Further more, the results of LEFSe analysis showed that OW disinfestation had different impacts on different fungi. The OW disinfestation had promoted the growth of *Trichoderma* and *Rhodotorula*, but it had inhibited the growth of *Hannaella*. A study suggested that *Trichoderma* and other beneficial microorganisms would help soil microbiome recover after soil fumigation disinfestation [24]. Also, it has been reported that *Trichoderma* could be used as the biocontrol fungi for plant disease prevention [25]. Not only that, it has also been documented that some *Trichoderma* strains could suppress pathogens like *F*. *pseudograminearum*, *M*. *syringejaponicae* and so on [26,27]. As for *Rhodotorula*, a research has suggested that one strain CAM4 of it could be used for salt and drought stress resistance biofertilizer [28]. Another study indicated that *Rhodotorula* strain CAH2 might be a plant growth promoting fungus in unfavourable environmental conditions [29]. Accordingly, OW disinfestation might promote the beneficial fungi growth during disinfestation. Notably, our findings indicated that the OW disinfestation could considerably relieve the soil deterioration of the continuous ginger field, whose effect was partly similar with bean dregs treatment [24]. However, many present soil disinfestation ways (i.e. reductive soil disinfestation and chemical soil disinfestation) have suppressed not only soil-borne pathogens but also fungal communities [1,24], which would alter the fungal community structure. In our study, non-pathogen fungal community has been reassembled and the growth of some non-pathogen fungi were promoted. Loganathachetti et al. have reported that there was a significant correlation between fungal community compositional changes and carbon or nitrogen availability of soil [30]. Therefore, our present study provided more insights into OW disinfestation research.

In conclusion, through a comprehensive analysis of the continuous ginger fields treated and untreated with OW, the results indicated that OW disinfestation had complicated impacts on fungal communities in continuous ginger fields. The OW disinfestation might promote the beneficial fungi growth while had a little negative influence on fungal communities in continuous ginger fields, which provided more reference information for soil disinfestation research.

## Supporting information

S1 Fig Venn diagrams of OTU changes of the CK group and O_3_ group. (A-E) The OTU changes were recorded at 0, 1, 3, 5 and 9 days. CK: control group; O_3_: The OW disinfestation treatment group.
(TIF)

S2 Fig The dilution curve of samples. **(A-E) The dilution curve at 0, 1, 3, 5 and 9 days.** CK: Control group; O_3_: The OW disinfestation treatment group. Five replicates in each group.
(TIF)

S3 Fig Microbial community relative abundance at genus level at 0, 1, 3, 5 and 9 days after OW disinfestation treatment and exposure to air. (TIF)

S4 Fig Venn diagrams of differential biomarkers at various days. **(A)** Venn diagram of the O_3_ group differential biomarkers at 0, 1, 3, 5 and 9 days. **(B)** Venn diagram of the CK group differential biomarkers at 0, 1, 3, 5 and 9 days. CK: Control group; O_3_: The OW disinfestation treatment group.
(TIF)
