# Supplementary figures and images for: Impact of ozonated water disinfestation on soil fungal community composition in continuous ginger field
Source: PLoS One. 2022 Apr 7;17(4):e0266619. doi: 10.1371/journal.pone.0266619 (PMC8989316; doi:10.1371/journal.pone.0266619)

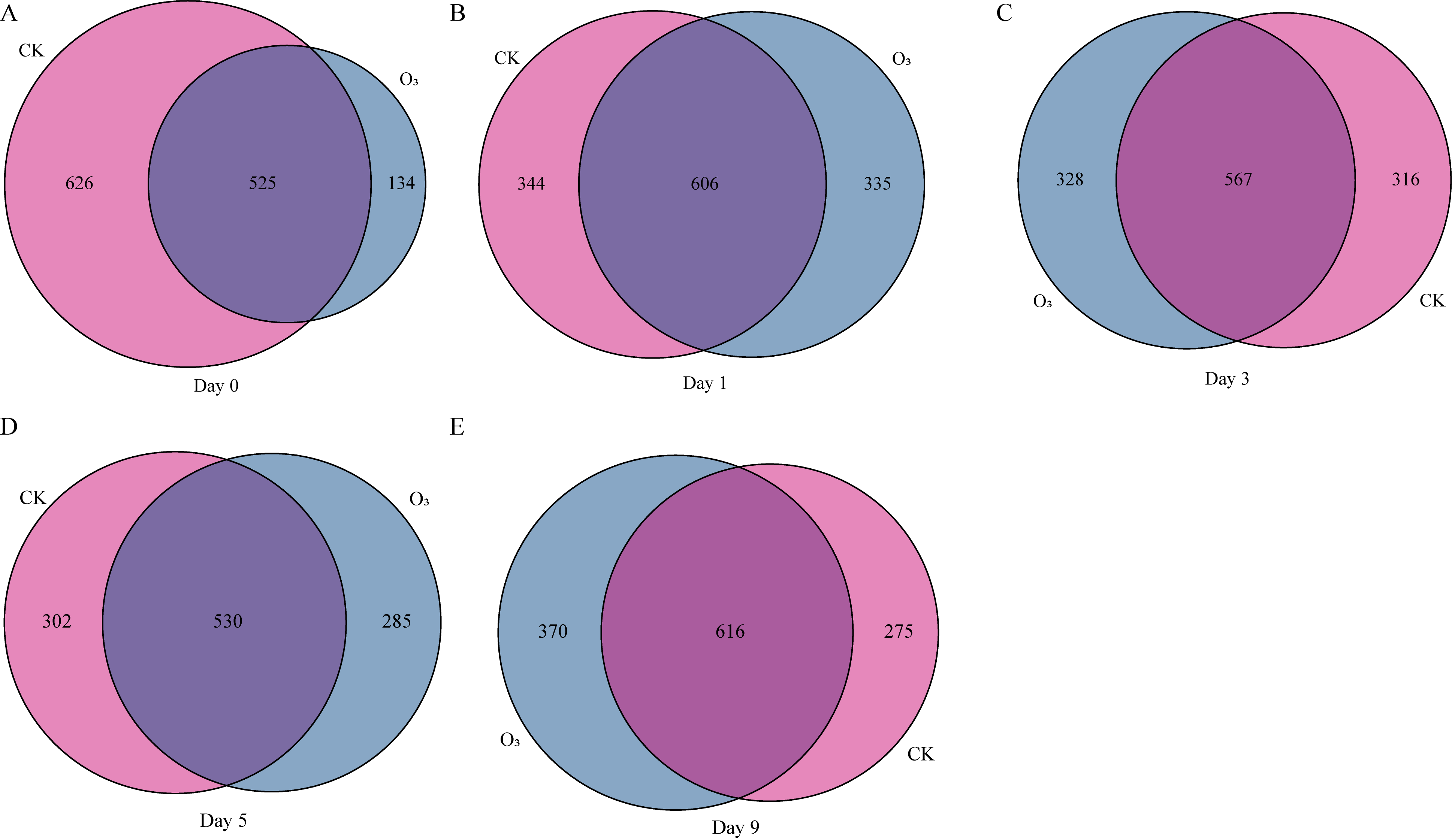

Supplement: S1 Fig — (A-E) The OTU changes were recorded at 0, 1, 3, 5 and 9 days. CK: control group; O3: The OW disinfestation treatment group. (TIF) [file pone.0266619.s001.tif]

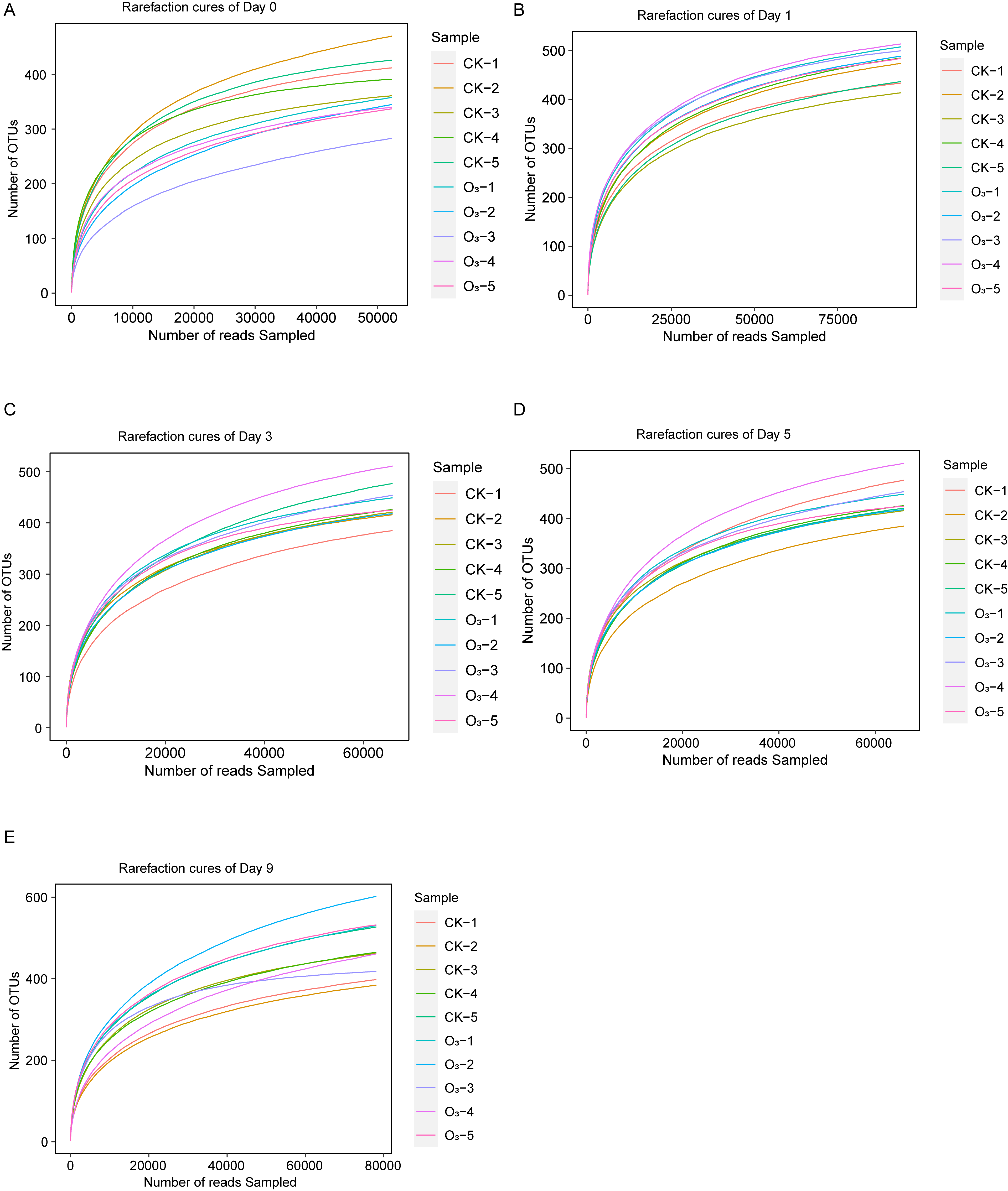

Supplement: S2 Fig — (A-E) The dilution curve at 0, 1, 3, 5 and 9 days. CK: Control group; O3: The OW disinfestation treatment group. Five replicates in each group. (TIF) [file pone.0266619.s002.tif]

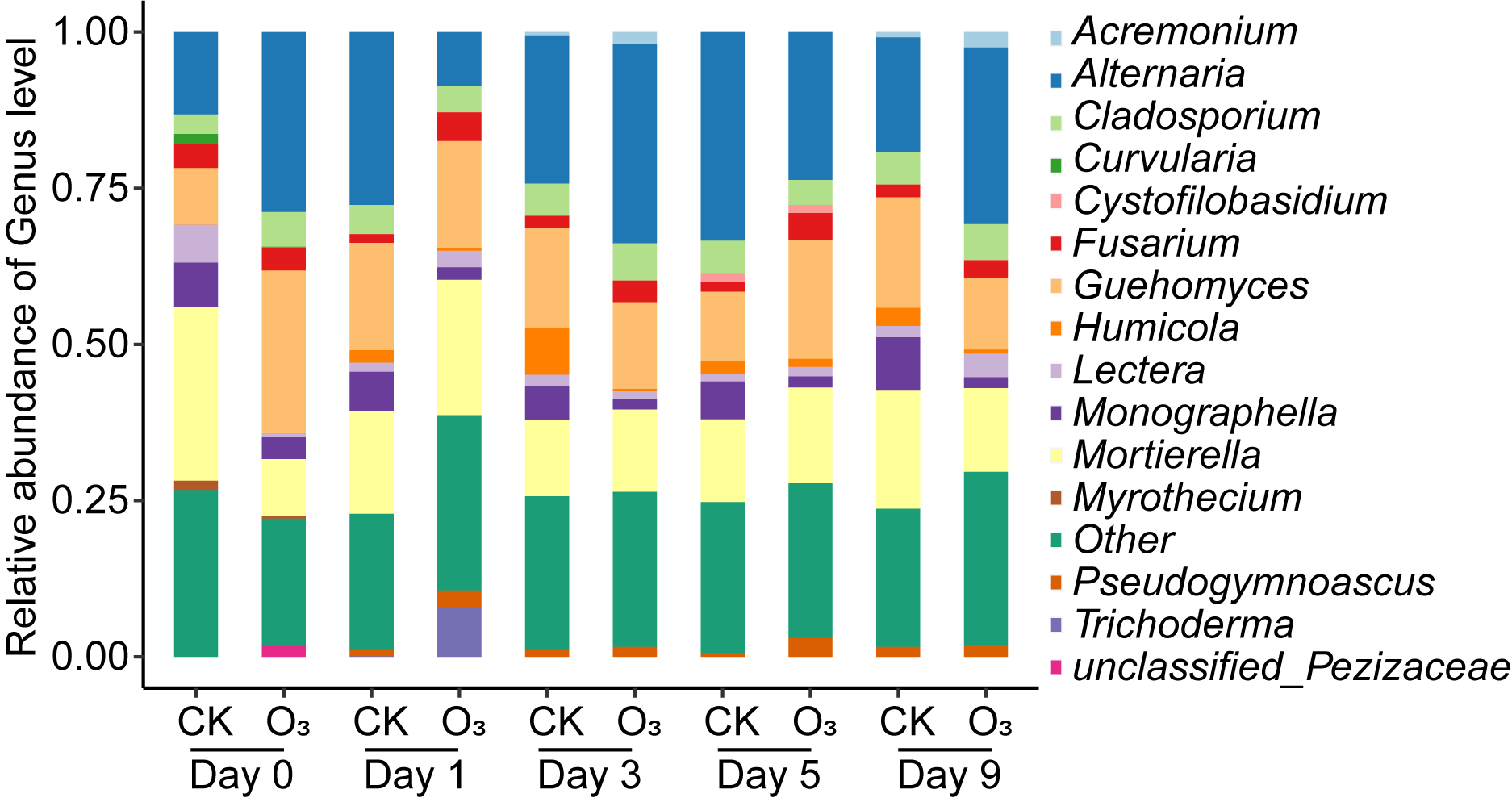

Supplement: S3 Fig — (TIF) [file pone.0266619.s003.tif]

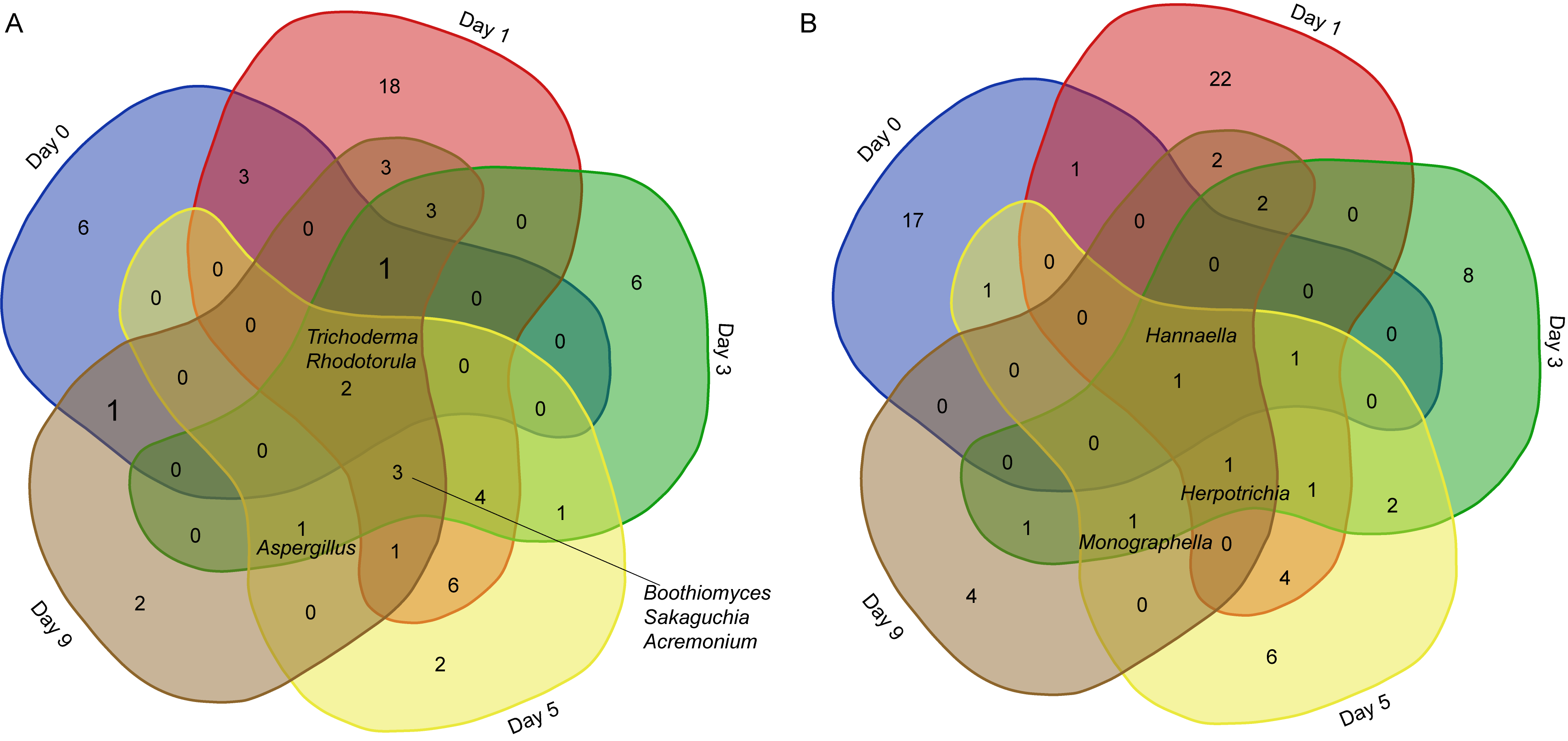

Supplement: S4 Fig — (A) Venn diagram of the O3 group differential biomarkers at 0, 1, 3, 5 and 9 days. (B) Venn diagram of the CK group differential biomarkers at 0, 1, 3, 5 and 9 days. CK: Control group; O3: The OW disinfestation treatment group. (TIF) [file pone.0266619.s004.tif]
